# Cell-surface GPNMB and induction of stemness

**DOI:** 10.18632/oncotarget.26472

**Published:** 2018-12-18

**Authors:** Yukari Okita, Chen Chen, Mitsuyasu Kato

**Affiliations:** Yukari Okita: Department of Experimental Pathology, Faculty of Medicine; Division of Cell Dynamics, Transborder Medical Research Center, University of Tsukuba, Ibaraki, Japan

**Keywords:** cancer stem cell, GPNMB, EMT, 3D cultures, sphere formation

Cancer tissue, like regenerating normal tissue, is composed of a heterogeneous cell population, but within that population, it contains a small proportion of cancer stem cells (CSCs). CSCs were first found in acute myeloid leukemia and later in solid tumors including those of the breast, brain, prostate, colon, pancreas, and lung. Owing to their dormancy, strong resistance to oxidative stress, abundant expression of multidrug exporters, and so forth, CSCs harbor the potential of tumorigenesis and of resistance to anti-cancer drugs or radiation. CSCs are thought to be the root cause of cancer metastasis and relapse. Therefore, therapy targeting CSCs is urgently needed to eradicate cancer. Since breast cancer stem cells (BCSCs) were isolated as cells with the markers of CD44^+^/CD24^-/low^/Lin^-^ [1], CD44^+^/CD24^-^ is frequently used as a BCSC marker. Recently, we have proposed a novel and potential BCSC marker, that is, cell surface-expressed Glycoprotein nmb (GPNMB) [2].

GPNMB is a type I transmembrane protein that is highly expressed in various cancers including breast cancer, especially in the most aggressive triple-negative type of breast cancer [3, 4]. Previously, we identified GPNMB as a target of the transcription factor MAFK and demonstrated that MAFK and GPNMB contribute to tumor initiation and malignant progression of TNBC cells through induction of epithelial-mesenchymal transition (EMT) [5]. We originally focused on MAFK because we found that it is a downstream molecule of transforming growth factor-β (TGF-β) signaling pathway, which has been well studied as an EMT inducer [6].

EMT is a biological process involved in embryonic development, wound healing, tissue fibrosis, and cancer invasion and metastasis [7]. Upon triggering of EMT, epithelial characteristics convert to mesenchymal ones; that is to say, cells lose their cell-cell adhesion and cell polarity and in turn acquire enhanced extracellular matrix formation and cell motility. Therefore, EMT is thought to promote cell invasiveness and cancer metastasis. During the past decade, attention has increasingly been focused on a link among EMT induction, stemness, and tumorigenesis. The concept of this link was originally reported in 2008 by Mani *et al.*, who showed that the EMT-related transcription factors (EMT-TFs) Twist and Snail as well as TGF-β1 induce the development of a CD44^+^/CD24^-^ BCSC population in mammary epithelial cells and that these cells actually have higher sphere-forming ability [8]. Although multiple reports have since clarified the roles of EMT-related molecules in the induction of CSC properties, the function of GPNMB in CSC induction has not been clarified [9, 10].

To address this issue, we enriched CSCs using a three-dimensional (3D) sphere-culture method and examined the correlation between GPNMB and CSC properties in breast cancer cells. The 3D culture system is thought to be better than the two-dimensional (2D) monolayer culture system at mimicking the cellular dynamics of the body and is used to enrich cells possessing stem cell properties in cancers of the breast, brain, and head and neck. Compared with the 2D cultures, the 3D cultures induced heterogeneity in the cell proliferation status, as well as higher expression of *GPNMB* and of the CSC genes *SOX2*, *NANOG*, *OCT4*, *CD44*, and *CD133*, the EMT-TF genes *SNAIL*, *SLUG*, and *ZEB1*, and the mesenchymal markers genes *CDH2*, *fibronectin*, and *vimentin*. In contrast, the expression of *CD24* and an epithelial marker gene, *CDH1*, decreased in the 3D cultures. We then isolated cell surface-GPNMB^high^ and -GPNMB^low^ cells from the 3D-cultured spheres. The cell surface-GPNMB^high^ cells expressed high levels of CSC and EMT-TF genes, had significantly higher secondary sphere-forming frequencies than did the cell surface-GPNMB^low^ cells, and showed no detectable levels of proliferation marker genes. However, the total GPNMB mRNA levels were comparable in the cell surface-GPNMB^high^ and -GPNMB^low^ cells. For this reason, we would like to emphasize the “cell surface” expression of GPNMB as a marker of CSCs although we do not yet fully understand the detailed sorting mechanism of GPNMB in dormant breast cancer cells [2].

Importantly, similar results were obtained from transplanted breast tumors. We found that the tumors contained both cell surface-GPNMB^high^ and -GPNMB^low^ cells and that the cell surface-GPNMB^high^ cells had higher expression of CSC and EMT-TF genes and showed higher secondary tumor-forming potential than did the cell surface-GPNMB^low^ cells. Additionally, further analysis of the tumors generated by the cell surface-GPNMB^low^ cells indicated that these tumors were also composed of a ratio of cell surface-GPNMB^high^ and -GPNMB^low^ cells comparable to that of the tumors made from cell surface-GPNMB^high^ cells, suggesting that the plasticity of cancer cells generates cell surface-GPNMB^high^ cells from cell surface-GPNMB^low^ cells at low frequency and causes the tumorigenic potential [2].

Furthermore, we examined the importance to CSC gene induction of the tyrosine residue in the hemi-immunoreceptor tyrosine-based activation motif (hemITAM) of GPNMB. When we replaced the tyrosine (Y) with phenylalanine (F), GPNMB lost its tumorigenic function [5]. The YF mutant, which lacks tumorigenic activity, did not induce the expression of CSC genes even in the 3D culture condition, indicating that stemness induction depends on the hemITAM function of GPNMB [2].

**Figure 1:**
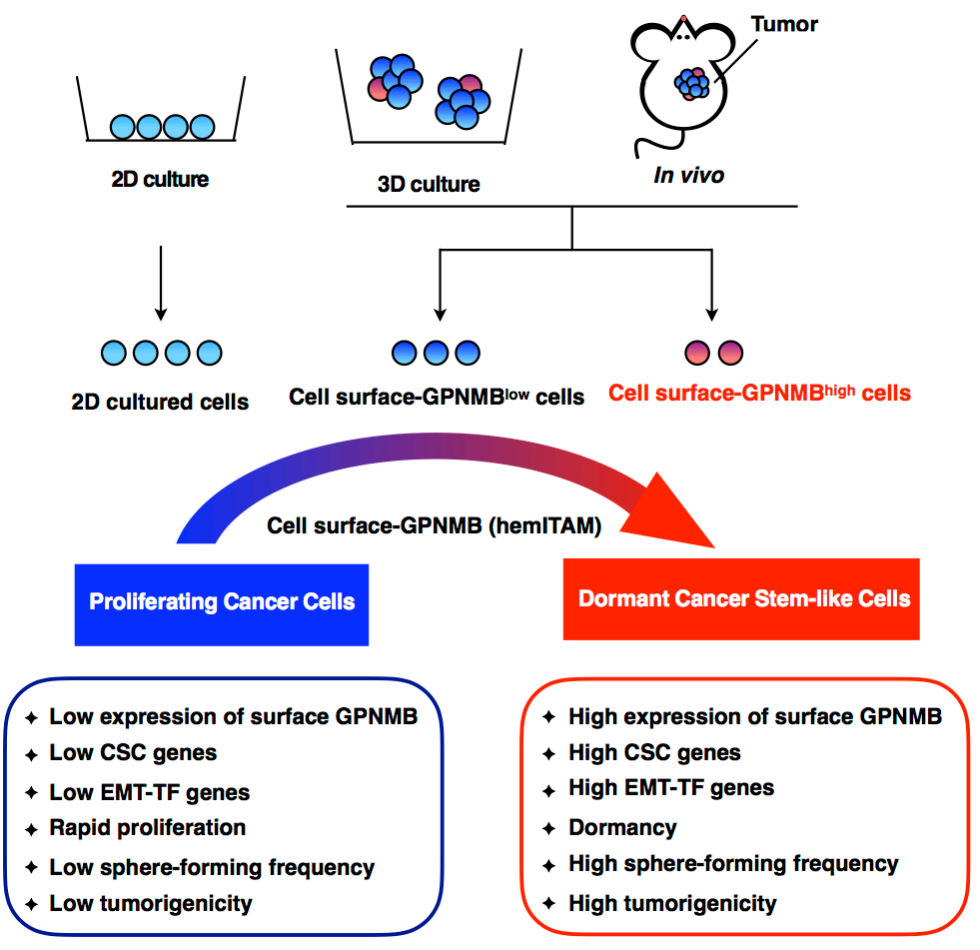
Schematic overview Characteristics of cell surface-GPNMB^low/high^ cells in 3D-cultured spheres and in *in vivo* tumors. When breast cancer cells are cultured in the 2D and 3D culture conditions or grown *in vivo*, cell surface-GPNMB^high^ cells are observed only in the 3D-cultured spheres or *in vivo* tumors. Compared with the cell surface-GPNMB^low^ cells, the cell surface-GPNMB^high^ cells show enhanced levels of CSC and EMT-TF genes, no detectable levels of proliferation marker genes, and higher frequencies of sphere/tumor formation depending on the tyrosine residue in hemITAM.

In summary, these novel findings suggest that GPNMB is exposed on the surface of dormant breast cancer cells, which contributes to the acquisition of stem cell-like properties through hemITAM and induces tumorigenic cell proliferation (Figure 1).
